# On improvement in ejection fraction with iron chelation in thalassemia major and the risk of future heart failure

**DOI:** 10.1186/1532-429X-13-45

**Published:** 2011-09-12

**Authors:** DJ Pennell, JP Carpenter, M Roughton, ZI Cabantchik

**Affiliations:** 1 Cardiovascular Magnetic Resonance Unit, Royal Brompton Hospital, London UK; 2 National Heart and Lung Institute, Imperial College, London UK; 3 Department of Statistics, University College London, UK; 4 Life Sciences Institute, Hebrew University of Jerusalem, Safra Campus-Givat Ram, Jerusalem, Israel

## Abstract

**Background:**

Trials of iron chelator regimens have increased the treatment options for cardiac siderosis in beta-thalassemia major (TM) patients. Treatment effects with improved left ventricular (LV) ejection fraction (EF) have been observed in patients without overt heart failure, but it is unclear whether these changes are clinically meaningful.

**Methods:**

This retrospective study of a UK database of TM patients modelled the change in EF between serial scans measured by cardiovascular magnetic resonance (CMR) to the relative risk (RR) of future development of heart failure over 1 year. Patients were divided into 2 strata by baseline LVEF of 56-62% (below normal for TM) and 63-70% (lower half of the normal range for TM).

**Results:**

A total of 315 patients with 754 CMR scans were analyzed. A 1% absolute increase in EF from baseline was associated with a statistically significant reduction in the risk of future development of heart failure for both the lower EF stratum (EF 56-62%, RR 0.818, p < 0.001) and the higher EF stratum (EF 63-70%, RR 0.893 p = 0.001).

**Conclusion:**

These data show that during treatment with iron chelators for cardiac siderosis, small increases in LVEF in TM patients are associated with a significantly reduced risk of the development of heart failure. Thus the iron chelator induced improvements in LVEF of 2.6% to 3.1% that have been observed in randomized controlled trials, are associated with risk reductions of 25.5% to 46.4% for the development of heart failure over 12 months, which is clinically meaningful. In cardiac iron overload, heart mitochondrial dysfunction and its relief by iron chelation may underlie the changes in LV function.

## Background

Major advances have been made in the last 10 years in the understanding and treatment of cardiac siderosis in beta-thalassaemia major (TM) patients. Those advances are attributable primarily to the development of a method for the quantification of myocardial iron based on magnetic resonance T2* imaging and its worldwide adoption,[1,2] which has permitted the recognition of the high prevalence of cardiac siderosis in patients chronically treated with deferoxamine, [1,3,4]the establishment of the association of cardiac siderosis with impaired left ventricular ejection fraction (LVEF) [1,5-7] the identification of iron-chelation regimes which are effective in removal of iron from the heart [8-12] and the reduction in cardiac mortality in populations[13-15]. Studies reporting on the use of iron chelators in treating transfusional iron overload have raised interesting questions with regard to cardiac efficacy relating to changes in the mean LVEF of the TM cohorts under study. Studies of TM patients in heart failure with severe cardiac siderosis and depressed LVEF show that combination treatment with deferiprone and deferoxamine increased mean absolute cohort LVEF by 14% over 1 year,[10] or treatment with continuous intravenous deferoxamine raised LVEF by 11%[16]. Changes in LVEF of this magnitude accompanied with relief of heart failure have clear clinical benefit. However, in TM patients with mild to moderate cardiac siderosis, randomized controlled trials of deferiprone monotherapy raised the mean cohort absolute LVEF by 3.1%,[8] whilst deferiprone in combination with deferoxamine raised absolute LVEF by 2.6%[9]. The clinical meaningfulness of these statistically significant but smaller improvements in LVEF is less clear. We therefore sought to determine the clinical significance of changes in LVEF from a UK database of TM patients by statistical modelling of the change in LVEF between serial scans to the future development of heart failure, which is an important clinical endpoint in TM associated with a high mortality[14,17].

## Methods

Ethical permission was obtained from the research ethical committee for this study. A database of 652 patients from 21 UK haematology centres was established and maintained prospectively, as previously reported[18]. We approached the question of the clinical significance of changes in LVEF by determining the number of patients recorded in the database who had: a) at least 2 cardiovascular magnetic resonance (CMR) scans; b) LVEF measured by CMR at each time point and a baseline EF of <71%, which is the lower half of the normal range for thalassaemia major patients without cardiac iron loading;[19] c) complete clinical follow-up for the clinical outcome of heart failure for 12 months after each scan. Patients on all treatments and all baseline cardiac T2* values were included. The patient demographics are shown in table 1. The primary outcome measure for this study was the development of symptomatic heart failure within 12 months of a CMR scan. A new diagnosis of heart failure was made only if the patient complained of worsening dyspnoea at rest or during exercise, objective LV dysfunction was present with an ejection fraction of <56%,[1,9] and the caring clinician made the clinical diagnosis of heart failure.

**Table 1:** Demographics of the patients at the first qualifying scan for analysis.

Baseline ejection fraction group	56%-62%	63%-70%
N	109	216
Age	26.6 (8.6)	26.6 (8.7)
Sex		
Male N (%)	59 (54.1)	110 (53.4)
Female N (%)	50 (45.9)	96 (46.6)
Ethnicity		
Indian N (%)	31 (28.4)	56 (27.2)
Greek N (%)	18 (16.5)	31 (15.1)
Cypriot N (%)	17 (15.6)	30 (14.6)
Pakistani/Bangladeshi N (%)	14 (12.8)	32 (14.8)
Turkish/Arabic N (%)	12 (11.0)	18 (8.3)
Italian/Romanian N (%)	6 (5.5)	12 (5.6)
Chinese/Malay N (%)	5 (4.6)	4 (2.0)
Unknown N (%)	7 (6.4)	20 (9.3)
Ferritin [ug/L]	2140 (1600)	2065 (1636)
Cardiac T2* [ms]	19.7 (13.3)	23.2 (13.8)
Liver T2* [ms]	4.5 (4.3)	5.1 (4.6)
LVEDV [mL]	132.1 (33.5)	128.3 (38.4)
LVESV [mL]	49.3 (19.9)	43.8 (19.0)
LVEF [%]	59.7 (1.8)	66.7 (2.3)
Treatment		
DFO N (%)	72 (66.1)	109 (52.9)
DFP N (%)	10 (9.2)	35 (17.0)
DFX N (%)	3 (2.75)	6 (2.9)
DFO + DFP N (%)	22 (20.2)	50 (24.3)
DFO + DFX N (%)	1 (0.9)	0 (0)
None N (%)	1 (0.9)	6 (2.9)

shown as mean (SD) except where N (%) is shown

LV-left ventricle, EDV-end-diastolic volume, ESV-end-systolic volume, EF-ejection fraction, DFO-deferoxamine, DFP-deferiprone, DFX-deferasirox.

Patients were scanned with a 1.5T scanner (Sonata, Siemens Medical Solutions, Erlangen, Germany) using previously reported techniques[20]. In brief, CMR was performed with a cardiac gated, single breath-hold, 8-echo sequence (2.6 to 16.7 ms, increasing in 2.02-ms increments) of a single mid-ventricular short-axis slice. Long-axis cines and a contiguous stack of short-axis cines were also acquired to assess left ventricular dimensions and function using standard techniques[21]. Data analysis was performed using CMRtools and its plug-in Thalassemia-Tools (Cardiovascular Imaging Solutions, London, UK) for the heart T2* (a large region of the interventricular septum excluding regions in proximity to the coronary veins), as well as left ventricular ejection fraction using semi-automated planimetry of endocardial borders[21]. All scans were reported at the time of acquisition by multiple operators, and a clinical report was generated for the referring physician.

### Statistics

All statistical analyses were performed by a medical statistician. Two categories of baseline EF were used in this analysis; 56-62% and 63-70%. These strata were used because 63% is the lower limit of normal for EF in thalassemia patients in the absence of myocardial siderosis,[19] and 56% is the lower limit of normal for EF in normal subjects,[21] and patients with EF below this are considered to have definite LV systolic dysfunction or heart failure. Patients were eligible for inclusion in either analysis provided they had one scan with an EF value within the range of either category. All subsequent scans for that patient were then analysed to assess the impact of the change in EF on the outcomes of heart failure. Since only the first scan in a series for each patient was required to be in the specified range, it is possible for patients to be included in both sets of analyses. The association between changes in EF (for each scan after the baseline scan, the change is calculated as the difference between the current scan and the baseline) and the outcomes was assessed using a mixed-effects Poisson regression model. Patients were entered as random effects in the model (the random effects are those things that we need to take account of, such as the repeated measures within patients, but which we do not want to estimate, for example we do not want to compare individual patients to one another) with the change in EF entered as a fixed effect (the fixed effect is the predictor variable which we explicitly want to estimate and quantify in terms of its association with the outcome). A p-value of < 0.05 was taken to be significant, and all analysis was performed using Stata 10.

## Results

**Figure 1:**
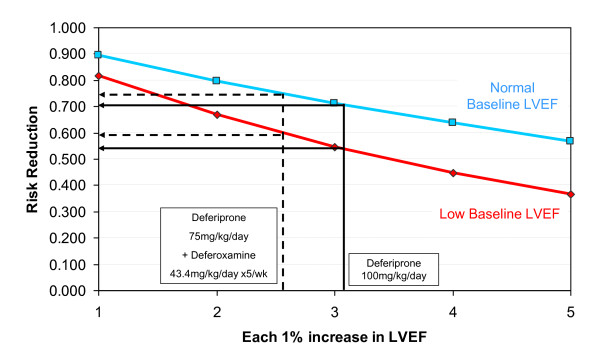
**Reduction in risk of heart failure**. Reduction in risk of heart failure shown for each 1% increase in ejection fraction for each baseline ejection fraction stratum of patients (blue- normal baseline EF; red- low baseline EF). The risk reduction associated with the increases in ejection fraction from 2 randomized controlled trials of deferiprone are shown, with the target drug doses[8,9]. The risk reductions associated with improvements in EF of 2-3% are substantial and clinically meaningful in both groups.

A total of 315 patients satisfied the selection inclusion criteria, who had undergone 754 CMR scans. There were 12 HF events in the 56-62% LVEF cohort and 14 in the 63-70% LVEF cohort. This equals an event rate of 11.0% and 6.8% respectively. Analysis of the occurrence of heart failure showed a relative risk of 0.818 (p < 0.001) for the stratum of patients with baseline EF 56-62%, which represents an 18.2% reduction in risk for each 1% increase in EF (table 2). In the stratum of patients with baseline EF of 63-70%, the relative risk was 0.893 (p = 0.001), which represents a 10.7% reduction in risk for each 1% increase in EF. The incremental effect of risk reduction associated with each 1% increase in EF associated with drug treatment is illustrated in Figure 1. The blue line shows the risk reduction for the group with normal baseline EF, and the red line shows the risk reduction for the group with low baseline EF. The overall risk reduction associated with the improvement in EF for 2 recent treatment trials is illustrated.

**Table 2:** Relative risk for development of heart failure for each 1% increase in ejection fraction (EF)

Baseline ejection fraction	Relative risk	95% Confidence intervals		p value	Patients	Scans
56-62%	0.818	0.767	0.872	<0.001	109	291

63-70%	0.893	0.836	0.953	0.001	206	463

## Discussion

Clinical trials of treatment frequently raise the challenging question of whether a statistically significant improvement in a trial endpoint is also clinically meaningful. This can only be addressed by examining the clinical outcomes associated with the improved endpoint, but trials are often designed for a technical outcome of efficacy which may leave them underpowered to directly derive the clinical meaningfulness. Such a situation has arisen in the first randomized clinical trials of oral iron chelation in cardiac siderosis of thalassemia major, where the endpoint of LVEF was shown to be significantly improved with treatment, but the sample size in the trials of approximately 60 patients and the period of follow-up were not sufficient to determine clinical outcomes[8,9]. Under these circumstances an alternative approach to the issue of assessing clinical significance is required. We have approached this issue by examining the clinical outcome of heart failure, with reference to changes over time in EF, in a database of TM patients which has sufficient size to answer outcomes questions. Statistical modelling was applied to a cohort of 315 patients who had at least 2 assessments of LV EF by CMR to establish the relative risk for development of heart failure within 12 months. In order to address the issue of the significance of changes in EF for patients whose baseline EF was in the normal range for thalassemia major in the absence of cardiac siderosis (>63%), we formed 2 strata for analysis related to normal ranges of EF, with baseline EF 56-62% (reduced EF for TM patients) and 63-70% (lower half of the normal range for TM patients). The results show that an increase in LVEF is associated with statistically significant risk reductions for heart failure for both strata of baseline EF. For a 1% increase in EF, the risk reduction for heart failure was 18.2% for the 56-62% EF stratum, and 10.7% for the 63-70% stratum, both of which are of substantial magnitude for such a small absolute change in EF.

These results can then be applied to the findings in 2 comparative cardiac treatment randomized controlled trials (Figure 1) which have been previously reported that have used state of the art measurement of EF with CMR, which is the most reproducible and accurate technique available[21-23]. For the deferiprone monotherapy randomized controlled trial, a statistically significant 3.1% mean increase in EF was observed in the deferiprone arm[8]. The data from the current study indicate that this degree of mean improvement in EF converts to a mean reduction in risk for development of heart failure of 46.4% and 29.6% for patients with a baseline EF of 56-62% and 63-70% respectively. For the combination deferiprone-deferoxamine randomised controlled trial, which showed a statistically significant EF increase of 2.6%,[9] the reduction in risk for development of heart failure is 40.7% and 25.5% for patients with a baseline EF of 56-62% and 63-70% respectively. The large magnitude of these risk reductions in heart failure indicate the statistically significant findings from the randomized controlled trials of deferiprone treatment are in addition, clinically meaningful. The results also indicate that there is a leftward shift in EF that occurs with cardiac iron loading, and that prognostically important subclinical LV dysfunction can be present with EFs in the lower normal range.

Although our analysis indicates significant clinical benefit from the observed improvements in EF seen in the randomized controlled trials of deferiprone, final confirmation can only be obtained from clinical studies comparing iron-induced cardiac morbidity and mortality in transfusion-dependent patients treated with the chelators for long periods of time. Randomized trials examining this question are not viable clinically, and the best alternatives therefore are observational reports of the long-term morbidity and mortality in association with iron chelators. A literature search revealed 6 publications that compared morbidity and mortality in deferiprone-treated and deferoxamine-treated patients. All studies reported significantly less morbidity and/or mortality with deferiprone, but none showed less morbidity or mortality in deferoxamine-treated patients[14,24-28]. Thus the predictions generated from the analyses in this current study are consistent with the clinical outcomes in large observational studies. The improved clinical outcomes are also consistent with the finding of higher EF with chronic deferiprone treatment compared with deferoxamine treatment in a matched cohort study from Anderson et al [29]. A further relevant study which is consistent with the importance of the EF for cardiac outcomes came from Davis et al, who studied 81 TM patients with no history of heart disease with long-term annual measurement of EF using radionuclide ventriculography[30]. Cardiac death was significantly more common in the patient cohort whose EF was <45% or who sustained >10% absolute fall in EF between consecutive measurements in comparison with patients whose EF was >45% and who had <10% absolute fall in EF between consecutive measurements. Although the absolute threshold for abnormal EF is lower for methodological reasons with the legacy radionuclide ventriculography technique than for the now widely available CMR method which was used in the current study, [31] the data of Davis et al confirms the importance of the maintenance of the resting EF in TM, and is in accord with the finding of the current study that a rise of EF is associated with a lower risk of developing heart failure.

Whilst the interpretation of these data indicates that small improvements in EF are associated with meaningful clinical benefits in outcomes, the mechanism of such benefit has not been elucidated. The benefit cannot be due to total body iron reduction, as deferiprone is no more effective than deferoxamine in this action [8]. There are also no data to indicate that deferiprone has any clinically significant inotropic or vasodilator effects, and thus it is unlikely that clinical benefit accrues from a primary effect on enhancing myocardial contractility or changing dynamics of vascular flow. On the other hand, the data do provide evidence of increased cardiac performance, as measured by changes in LVEF. Thus it is reasonable to consider mechanisms that might enhance contractility in a heart that is beginning to fail in patients with transfusional iron overload, and which could be altered by the administration of an iron chelator like deferiprone.

Considering possible mechanisms that might enhance contractility in the failing heart of patients with transfusional iron overload we can draw some analogies with recent findings in Friedreich's Ataxia, particularly the association of cardiac abnormalities with excessive mitochondrial iron accumulation and their correction by iron chelation [32]. We hypothesize that iron-mediated damage of the heart, as reflected in LV function in iron overload, is associated with heart cell mitochondria[33]. The endosymbiont mitochondria with their own DNA are subcellular organelles which distinguish eukaryotic from prokaryotic cells, and drive the cellular bioenergetics responsible for the genomic complexity that has permitted the evolution of multicellular organisms[34]. Mitochondria generate cellular energy in a biologically useful form in the form of adenosine triphosphate (ATP), and in the healthy human heart, mitochondria harness more than 90% of cell ATP production via oxidative phosphorylation[35]. In disease states such as heart failure,[36] the mitochondrial oxidative capacity of cardiac muscle cells may be diminished,[37-43] and consequently the contractile performance of the failing heart may be limited. In systemic iron overload, mitochondria are the major cellular sites of toxic iron accumulation,[44] and it is therefore sensible to consider mitochondrial "toxic iron" as a major pathophysiological factor and an important target for iron chelation[45]. Evidence that heart failure in chronic iron overload may be largely due to mitochondrial damage [46] was obtained in mice injected with iron dextran over 4 weeks[47]. These animals showed various pathophysiological changes due to chronic iron-induced organ toxicity, including cardiomyopathy: a) total cardiac iron was increased almost 10-fold, which is similar in magnitude to that reported at autopsy in hearts of TM patients who had died of heart failure [2]; b) iron analysis revealed approximately a 50% increase in extractable (trichloroacetic acid-soluble) cardiac iron in iron-injected animals; c) both biochemical and electron microscopy analyses revealed extensive damage to mitochondria in myocytes of iron-treated mice. The loss of mitochondrial respiratory capacity and ensuing cardiac dysfunction were attributed to decreased expression of mitochondria encoded mRNA and proteins due to mitochondrial DNA damage[47].

This leads to the question of why the heart is particularly sensitive to excessive iron loading compared to other tissues of polytransfused patients, which, in fact, might accumulate even larger amounts of the metal. The heart may be particularly sensitive to iron-induced mitochondrial damage relative to other tissues because of the large number of mitochondria which are required for its high respiratory requirements, and its low level of antioxidants[48]. Mitochondria generate ATP as a result of the action of 5 respiratory chain complexes,[49] and it has been estimated that the human heart creates 6 kg of ATP daily for its metabolic needs[50,51]. In human heart, deranged high energy phosphate metabolism is detectable in heart failure and is a predictor of mortality[52]. In rat heart cells, iron exposure reduced enzyme complex activity by up to 65%, which resulted in approximately a 25% drop in ATP production[49]. Contractility is probably downregulated in response to the reduced ATP production, and steady state ATP levels are maintained until a more end-stage situation. Respiratory chain inhibition could result from 4 main mechanisms: First, iron exposure increases the generation of toxic reactive oxygen species (ROS),[53] especially hydroxyl radicals,[54] which can lead to peroxidation of lipid components of the mitochondrial membrane such as cardiolipin, and impairment of respiratory chain complex assembly and activity;[55,56] Second, peroxidation of polyunsaturated fatty acids can lead to covalent protein linking which alters enzyme activity;[57] Third, iron may bind directly to respiratory chain enzymes and create active oxygen species which convert amino acid residues to carbonyl derivatives that reduce catalytic activity and increase protein degradation;[58] Fourth, ROS cause cumulative damage to mitochondrial DNA, reduced mitochondrial mRNA and impaired synthesis of respiratory chain subunits coded by the mitochondria, whereas subunits coded by nuclear DNA are unaffected[33,47]. A vicious cycle may be created where the respiratory chain enzymes may undergo oxidative inactivation which may lead to incomplete reduction of molecular oxygen and increased formation of free radicals[59]. ROS and mitochondrial dysfunction have also been linked to activation of the NLRP3 inflammasome, which is a molecular platform that triggers innate immunity and pro-inflammatory cytokines[60]. ROS also cause opening of the mitochondrial permeability transition pore, which can lead to cardiomyocyte apoptosis [61,62]. These cellular and systemic mechanisms associated with iron and redox dyshomeostasis might underlie the catastrophic deterioration in cardiac function that occurs as a terminal event in thalassemia major patients with heart failure.

Regardless of which of the above mechanisms is primarily responsible for most of the iron-ROS evoked damage in transfusional iron overload, improving EF and meeting the high metabolic demands for increased cardiac muscle contraction would depend on the ability to restore mitochondrial activities and thereby improve myocardial cell function. As increased cell labile iron levels might play a key role in the pathophysiology of heart failure,[63] a rational approach for treating cardiac iron overload would entail reduction of intracellular labile iron levels. An ideal iron chelating agent for this would readily cross cellular and subcellular membranes, and have an adequate affinity for specifically binding labile iron while forming non-redox active iron complexes that can also exit cells but also minimally interfere with resident cell activities, particularly those that are iron-dependent. Among the chelators in clinical use, deferiprone was found to optimally meet the above criteria in a frataxin-deficient HEK-293 cell model of mitochondrial iron accumulation [64]. Recent trials with deferiprone on Friedriech's ataxia patients indicate that such a modus operandi might also be operative in the clinical setting of hypertrophic cardiomyopathy observed in these patients[32].

The three clinically available iron chelators, deferoxamine, deferiprone and deferasirox all remove iron from the body, but display different efficacy in removing iron from different organs. Some of these differences are attributable to their different chemical structures that confer them with different chemical and physicochemical properties resulting in different bioavailability, pharmacokinetics and tissue distribution including accessibility to cellular labile iron pools [65-67]. The small molecular weight bidentate deferiprone, combined with its neutral charge and optimal partition coefficient displays greater ability to permeate membranes, [68,69] and remove iron from the myocardium than the much larger, charged hexadentate deferoxamine molecule[70]. The recently reported longitudinal 12 month trial of deferasirox in asymptomatic myocardial siderosis also showed good cardiac iron removal, but with an absence of improvement in LVEF,[11] with both effects persisting to 2 years,[71] and 3 years[72]. These results were very similar to those found in the US04 study[73,74].

Possible differences in the mode of action of deferiprone versus deferasirox might relate to a differential drug accessibility to labile iron pools within cells (particularly within mitochondria) or with an intrinsic ability of a chelator to associate with cell components such as membranes or proteins and thereby become retained in cells. Prolonged retention in cells can also be deleterious as a chelator can interfere with the biosynthetic machinery of heme or iron-S-clusters, and in extreme conditions could drive cells into an apoptotic path[75-77]. On the other hand, intermittent application of high doses of deferasirox to aged animals on alternate days was not only effective in reducing age related iron accumulation but also iron-mediated cell apoptosis[78]. One can argue that a relatively short time of tissue exposure to a chelator might limit iron chelation efficacy in the organism. However, relatively short exposures might also be advantageous as possible interferences with essential iron-dependent processes are also reduced. We propose that this "kiss-and-go" modality of chelation might be appropriate for removing iron that slowly accumulates in particular cell compartments until reaching toxic levels, as supported by basic and clinical data[64,79-81]. Further investigation of these possibilities is required to explain the intriguing differences in ejection fraction responses among the iron chelators. Whilst further basic science studies will undoubtedly be helpful in this mechanistic dissection, newer clinical trials may also yield clues. For example, as clinical experience grows in combining deferasirox with deferiprone for patient treatment,[82] it would be interesting to identify whether this combination is better, similar or worse at improving LVEF in patients with stable chronic cardiac iron loading than deferiprone in isolation.

## Conclusion

Analysis of a large TM database showed that small changes in ejection fraction have statistically significant and clinically meaningful effects in reducing the important clinical outcome of heart failure. These data suggest that the results from randomized controlled trials using deferiprone in TM yield a 40.7% to 46.4% relative risk reduction for heart failure in patients with a baseline ejection fraction below normal of 56-62%, and a 25.5% to 29.6% relative risk reduction for heart failure in patients with normal baseline ejection fraction of 63-70%. These predicted improved outcomes are consistent with several reports of reduced heart disease and increased survival in transfusion-dependent patients treated with deferiprone, lending support to the predictive value of improvement in clinical outcome as a consequence of increasing LVEF. The data indicate that prognostically important subclinical LV dysfunction can occur in TM patients with cardiac iron overload whilst EF remains in the lower normal range. The most likely explanation for the improvement in LV function found in the randomized controlled trials using deferiprone, is improved myocardial mitochondrial function although the exact mechanisms remain to be fully elucidated.

## Competing interests

Dr Pennell is a consultant to Siemens, Novartis and Apotex, and has received research support and speaker's honoraria. He is a director of Cardiovascular Imaging Solutions. John-Paul Carpenter and Michael Roughton have no conflicts to declare. Dr Cabantchik has received research support and speaker's honoraria from Novartis and Apotex.

## Authors' contributions

DJP conceived the study and wrote the manuscript. JPC was responsible for data collection. MR performed the statistical analysis. IZC assisted in interpretation of the results and basic science discussion of mitochondria. All authors read and approved the final manuscript.
